# Overcoming obstacles in Panama to starting a renal biopsy program in a rural area during the COVID-19 pandemic

**DOI:** 10.1007/s40620-022-01403-z

**Published:** 2022-08-23

**Authors:** Karen Courville, Rolando Milord, Jonathan Cerrud, Norman Bustamante

**Affiliations:** 1 Section of Nephrology, Department of Medicine, Hospital Dr. Gustavo Nelson Collado, Chitré, Herrera Panama; 2 Instituto de Ciencias Médicas, Las Tablas, Los Santos Panama; 3 Department of Pathology, Universidad de Panamá, Panama City, Panama; 4 Section of Interventional Radiology, Department of Radiology, Hospital Dr. Gustavo Nelson Collado, Chitré, Herrera Panama

**Keywords:** Kidney biopsy, Panama, COVID-19, Nephrotic syndrome, Nephritic syndrome

## Abstract

Due to the many implemented restrictions, the SARS-CoV-2 pandemic has rendered some tasks more difficult, for instance, the evaluation of outpatients. Panama’s tertiary care hospital for kidney biopsy referral was transformed into a COVID-only hospital in order to assist the large number of COVID-19 patients. In order to face the impossibility of following patients with nephrotic or nephritic syndrome, a biopsy program was implemented in a southern province in Panama. Thirty kidney biopsies were carried out over a 1-year period. This experience shows that kidney biopsy programs, that are usually run only in large referral centers, can also be implemented in small nephrology centers, allowing to obtain accurate diagnoses and to guide correct treatment.

Diagnosis and care of renal disease in developing countries is fraught with obstacles. The COVID-19 pandemic made some tasks even more difficult, for example outpatient kidney care during 2020 in Panama, due to disruption of the usual network of care, and the reassignment of health personnel to COVID rooms or vaccination posts [1].

Panama has a fragmented health care system: the Ministry of Health of Panama is responsible for regulating all health care activities and for providing care to the non-working population. Social Security is a semi-autonomous entity that provides services to the employed population. Ninety percent of the population uses these services, since there are cross-subsidies. A small percentage of the population, 10%, uses only private insurance.

During the pandemic, the National Referral Hospital, a third-level hospital that is part of the Social Security network where kidney biopsies were performed, stopped providing care for outpatient evaluation and elective procedures because it became a COVID hospital. The provinces of Herrera and Los Santos are considered rural and thus have a single second level reference hospital with two nephrologists to provide care for a population of 200,000 individuals. Renal biopsies were not performed at this institution.

Data reported in 2009 by the National Referral Hospital where renal biopsies were performed, and by the Social Security registries for 2020 showed that 66 kidney biopsies were performed over the first half of that year. In 2021, the number of procedures increased to 170 biopsies [2].

In 2020, as patients could not be referred for a kidney biopsy in Panama City, it was decided to request authorization from the medical board to implement a local renal biopsy program.

Physicians were trained and the biopsies were performed according to good clinical practices and international guidelines [3]. The patient’s therapy was reviewed and it was ascertained that the patient did not have coagulation abnormalities. All patients were hospitalized on the day of the procedure and discharged the next morning if no complications occurred.

Two cores were obtained from most patients, except in cases of poor tissue sample in immediate evaluation at light microscopy (less than 10 glomeruli) for which a third core was obtained. One core per patient was immediately stored in 10% buffered formalin for light microscopy and the other one in Michel’s transport media for immunofluorescence [4]. All samples were labeled with the patient’s name and identification number and put into a transport cooler to maintain an adequate temperature, (Panama has an average temperature over 30 °C most of the year) while waiting to be transported to the Nephropathology center the next morning, 250 km away in the Referral Hospital. After the biopsy the patient rested for 4 h, arterial pressure was checked according to the standard procedure, urine was evaluated and hemoglobin was checked 4 h later. [5]

Table 1 shows the characteristics of our 30 patients from March 2021 to March 2022. The information was recorded in paper-based records in the Hospital.

**Table 1:** Patients’ demographic characteristics, hemoglobin and blood pressure values

*n*	30	
Male (%)	53	
	Mean	SD ±
Age (years)	40.27	± 16.87
Hb pre biopsy (g/dL)	12.94	± 2.02
Hb post biopsy (g/dL)	12.33	± 2.15
SBP (mmHg) pre biopsy	126	± 9.77
DBP (mmHg) pre biopsy	79.17	± 6.17
SBP (mmHg) post biopsy	126	± 15.17

*Hb* hemoglobin, *SBP* Systolic Blood Pressure, *DBP* Diastolic Blood Pressure, *SD* Standard Deviation.

At the time of biopsy, 40% of the patients presented a nephrotic syndrome and 20% a nephritic syndrome. The biopsies were crucial to determine the prognosis and treatment for these patients. For example, one patient with chronic kidney disease of unknown origin was diagnosed with chronic interstitial nephritis, compatible with Mesoamerican Nephropathy. The region is an important agricultural production area, so this is a disease increasingly observed in this part of the country. Other findings are shown in Table 2.

**Table 2:** Pathology findings in renal biopsies

	Frequency	%
Focal and segmental glomerulosclerosis	5	16.7
Crescentic glomerulonephritis	4	13.3
IgA nephropathy	3	10.0
Lupus Nephritis 3 + 4 + 5	4	10.0
Membranous nephropathy	2	6.6
Cyclosporine toxicity	2	6.6
mTOR toxicity	1	3.3
Chronic graft Rejection	1	3.3
Global Glomerulosclerosis	1	3.3
Normal kidney	1	3.3
Renal Sarcoidosis	1	3.3
Minimal change disease	1	3.3
Renal amyloidosis	1	3.3
Post-infectious glomerulonephritis	1	3.3
Chronic Interstitial Nephritis or Mesoamerican Nephropathy	1	3.3
Normal Kidney Graft	1	3.3
Total	30	100.0

No major complications occurred: one patient presented self-limiting macroscopic hematuria and one patient presented local pain that improved with analgesia. This patient underwent a control ultrasound, which revealed a subcapsular hematoma of small size with no hemodynamic instability. Complete resolution was observed.

Although the biopsies had to be transported 250 km to reach the nephropathology center, the tissue was well-preserved and the various stainings could be carried out without problems. This program has helped patients save on the transportation costs of traveling, and allowed to tailor kidney care interrupted by the pandemic.

Kidney biopsy is often considered as highly specialized procedure, and is often performed in only a few settings. Under the pressure of need, during the COVID-19 pandemic, we have learned that kidney biopsy programs could be implemented in small nephrology centers in rural areas, following guidelines and safety precautions, with the support of referral Hospitals, thereby saving costs to patients and improving diagnosis and treatment for patients who live far from tertiary Centers.

## References

[1] Loutradis C, Pitoulias AG, Pagkopoulou E, Pitoulias GA (2021). Cardiovascular complications after COVID-19 in chronic kidney disease, dialysis and kidney transplant patients. Int Urol Nephrol.

[2] Giraldo E, Williams R, Bustamante N, Courville K, Viggiano C, Milord R. Estadísticas de biopsias renales realizadas en el Complejo Hospitalario Dr. Arnulfo Arias Madrid. Panamá 2005–2007. IV Congreso Nacional de Nefrología e Hipertensión 18 y 19 de septiembre de 2009, Cuidad de Panamá.

[3] Hogan JJ, Mocanu M, Berns JS (2016). The native kidney biopsy: update and evidence for best practice. Clin J Am Soc Nephrol.

[4] Fogo AB (2003) Approach to renal biopsy. Am J Kidney Dis 42(4):826–836. https://pubmed.ncbi.nlm.nih.gov/14520635/14520635

[5] Šimunov B, Gunjača M, Čingel B, Škegro D, Knotek M (2018). Safety of outpatient kidney biopsies. Nephron.

